# Noradrenergic regulation of skeletal muscle oxygen pressures: Impact of heart failure with preserved ejection fraction and heat therapy

**DOI:** 10.1113/EP092867

**Published:** 2025-07-20

**Authors:** Edward T. N. Calvo, Jacob M. Pontorno, Benjamin Zeidler, Taciane M. M. Pejon, Michael D. Belbis, Scott K. Ferguson, Craig J. Goergen, Timothy P. Gavin, Bruno T. Roseguini, Igor A. Fernandes, Daniel M. Hirai

**Affiliations:** ^1^ Department of Health and Kinesiology Purdue University West Lafayette Indiana USA; ^2^ Weldon School of Biomedical Engineering Purdue University West Lafayette Indiana USA; ^3^ Department of Physiological Sciences Federal University of São Carlos São Carlos SP Brazil; ^4^ Department of Exercise Science Aurora University Aurora Illinois USA; ^5^ Department of Human Factors and Behavioral Neurobiology Embry‐Riddle Aeronautical University Daytona Beach Florida USA

**Keywords:** exercise, functional sympatholysis, heart failure with preserved ejection fraction, microcirculation, obese ZSF1

## Abstract

Attenuation of sympathetic vasoconstriction during exercise (functional sympatholysis) contributes to skeletal muscle oxygen delivery–utilization matching. However, the extent to which muscle contractions impact noradrenergic regulation of interstitial oxygen pressures (${P_{O_{2}}}_{\mathrm{is}}$; the driving force for blood–myocyte oxygen flux) is unknown. We tested the hypotheses that (1) muscle contractions would attenuate the noradrenaline‐induced reduction in muscle ${P_{O_{2}}}_{\mathrm{is}}$ compared to rest (thus indicating functional sympatholysis) in healthy rats, and (2) functional sympatholysis would be impaired in rats with heart failure with preserved ejection fraction (HFpEF) but ameliorated with heat therapy. Skeletal muscle ${P_{O_{2}}}_{\mathrm{is}}$ was determined via phosphorescence quenching in anaesthetized healthy (Sprague–Dawley, *n* = 14) and HFpEF rats (obese ZSF1, *n* = 20) at rest and during contractions following noradrenaline superfusion (5 × 10^−4 ^ M). HFpEF rats underwent 8 weeks of heat therapy (HEAT, *n* = 10) or control treatment (CON; *n* = 10). Functional sympatholysis was evaluated based on the noradrenaline‐induced changes in ${P_{O_{2}}}_{\mathrm{is}}$ at rest and during contractions normalized to mean arterial pressure (Δ${P_{O_{2}}}_{\mathrm{is}}$/MAP; %/mmHg). Consistent with our hypothesis, muscle contractions attenuated the noradrenaline‐evoked ${P_{O_{2}}}_{\mathrm{is}}$ reductions in healthy rats (rest: −0.50 ± 0.23, contractions: −0.25 ± 0.16; *P *< 0.05). Compared to healthy rats, the noradrenergic response at rest was exacerbated in HFpEF‐CON (−0.85 ± 0.13; *P *< 0.05) but restored in HFpEF‐HEAT (−0.61 ± 0.25; *P *> 0.05). During contractions, the noradrenergic response was not different between HFpEF‐CON and HFpEF‐HEAT (−0.94 ± 0.07 and −0.86 ± 0.09, respectively; *P *> 0.05). Moreover, the magnitude of sympatholysis was lower in both HFpEF‐CON and HFpEF‐HEAT compared to healthy. Taken together, these results indicate that heat therapy failed to improve functional sympatholysis in HFpEF rats but restored the noradrenergic response in resting skeletal muscle.

## Abstract (highlights)

- **What is the central question of this study?**
  We evaluated (1) the noradrenergic regulation of skeletal muscle oxygen pressures in health and heart failure with preserved ejection fraction (HFpEF), and (2) whether heat therapy improves functional sympatholysis in HFpEF.
- **What is the main finding and its importance?**
  HFpEF rats exhibited exaggerated noradrenergic responses at rest and during contractions. Heat therapy did not ameliorate functional sympatholysis in HFpEF but restored the resting noradrenergic response. These observations provide novel insights into muscle dysfunction and plasticity in HFpEF and suggest that heat therapy might represent a useful adjunct intervention for this patient population.

## 1 INTRODUCTION

Skeletal muscle contractions require a highly coordinated neurovascular response to match tissue oxygen delivery and utilization (Joyner & Casey, 2015; Laughlin et al., 2012). Among many mechanisms, attenuation of sympathetic vasoconstriction during exercise (i.e., functional sympatholysis) plays a key role in the regulation of arterial blood pressure, peripheral O_2_ delivery and exercise tolerance (Joyner & Casey, 2015; Laughlin et al., 2012; Remensnyder et al., 1962). The magnitude of sympatholysis can be determined using muscle bulk blood flow (Doppler ultrasound) or tissue oxygenation (near‐infrared spectroscopy; NIRS) responses to sympathoexcitatory stimuli (e.g., phenylephrine, noradrenaline (NA) or tyramine infusions; lower‐body negative pressure; cold pressor test; nerve stimulation) at rest and during muscle contractions (Chavoshan et al., 2002; Fadel et al., 2004; Hansen et al., 1996; Hart et al., 2011; Kneale et al., 2000; Sprick et al., 2019; Teixeira et al., 2022; Vongpatanasin et al., 2011; Wray et al., 2007). Fadel et al. (2004) demonstrated that changes in NIRS‐derived muscle oxygenation to direct nerve stimulation reflect sympathetic vasoconstriction in anaesthetized rats. However, the extent to which muscle contractions impact noradrenergic regulation of interstitial oxygen pressures (${P_{O_{2}}}_{\mathrm{is}}$) is unknown. This is important because, as dictated by Fick's law, ${P_{O_{2}}}_{\mathrm{is}}$ provides the exclusive driving force for oxygen diffusion into the myocyte, thereby supporting oxidative phosphorylation (Hirai et al., 2018; Hirai, Colburn, et al., 2019). Within this context, skeletal muscle ${P_{O_{2}}}_{\mathrm{is}}$ measurements in preclinical models could help elucidate the impact of disease and treatment on functional sympatholysis.

Heart failure with preserved ejection fraction (HFpEF) is a debilitating clinical syndrome with high morbidity, mortality and healthcare costs (Martin et al., 2025). The hallmark symptom of HFpEF is severe exercise intolerance (Pandey et al., 2021; Poole et al., 2012). Although multiple mechanisms underlie impairments in physical capacity, skeletal muscle abnormalities are now recognized to be intrinsic to the HFpEF syndrome (Kitzman et al., 2017; Pandey et al., 2021; Poole et al., 2012). Recent evidence implicates impaired functional sympatholysis in reduced contracting muscle oxygen delivery in HFpEF patients (Alpenglow et al., 2024, 2023). However, to date, few therapeutic options exist to combat skeletal muscle abnormalities and ameliorate physical capacity in HFpEF (Heidenreich et al., 2022; Pandey et al., 2021; Shah et al., 2020).

Heat therapy has emerged as a practical intervention to improve cardiovascular health in young individuals and clinical populations (Brunt & Minson, 2021; Kim et al., 2020). Foundational work by Tei and colleagues (Kihara et al., 2002; Kisanuki et al., 2007; Miyata et al., 2008; Tei et al., 1995) revealed that repeated heat therapy improves multiple clinical and physiological outcomes in patients with heart failure with reduced ejection fraction (HFrEF). These and other studies (Ohori et al., 2012; Sobajima et al., 2015) reported that heat therapy improved endothelial function, cardiac structure and function, exercise tolerance, and quality of life in HFrEF patients. While the benefits of heat therapy are well documented in HFrEF, its efficacy and mechanisms of action in HFpEF remain largely unexplored. Our previous studies indicated, for the first time, that heat therapy can lead to central and peripheral adaptations that improve exercise tolerance in a preclinical model of HFpEF (Belbis et al., 2025). Nonetheless, whether heat therapy improves functional sympatholysis in HFpEF remains to be determined.

The purpose of this study was two‐fold. First, to determine the effects of topical noradrenaline on resting and contracting skeletal muscle ${P_{O_{2}}}_{\mathrm{is}}$ in healthy rats. We tested the hypothesis that muscle contractions would attenuate the noradrenaline‐induced reduction in tissue ${P_{O_{2}}}_{\mathrm{is}}$ compared to rest, indicative of functional sympatholysis. Second, to evaluate the effects of heat therapy on functional sympatholysis in a preclinical model of HFpEF. We hypothesized that functional sympatholysis would be impaired in HFpEF rats (evidenced by blunted reductions in contracting muscle ${P_{O_{2}}}_{\mathrm{is}}$ upon noradrenaline administration) but ameliorated with heat therapy. Confirmation of these hypotheses would support that (1) a novel combination of muscle ${P_{O_{2}}}_{\mathrm{is}}$ measurements and superfusion techniques can be used to evaluate functional sympatholysis in intact rat skeletal muscle, and (2) this preparation can detect alterations in functional sympatholysis produced by disease as well as therapeutic interventions.

## 2 METHODS

### 2.1 Ethical approval

All procedures and protocols were approved by the Purdue University Institutional Animal Care and Use Committee (protocol no. 2005002038) and followed guidelines established by the National Institutes of Health. We adhered to *Experimental Physiology*’s policies regarding animal experiments. Rats were maintained in accredited facilities (Association for the Assessment and Accreditation of Laboratory and Animal Care) under a 12:12 h light–dark cycle (lights on at 06.00 h and off at 18.00 h) with food and water provided ad libitum.

### 2.2 Experimental groups

A total of 34 rats were obtained from Charles River Laboratories (Boston, MA, USA) for this investigation. In Part A, the effects of NA superfusion on resting and contracting spinotrapezius muscle ${P_{O_{2}}}_{\mathrm{is}}$ were determined in healthy male Sprague–Dawley rats (∼2–3 months old; 356 ± 17 g; *n* = 14). In Part B, the effects of NA superfusion on resting and contracting spinotrapezius muscle ${P_{O_{2}}}_{\mathrm{is}}$ were determined in a pre‐clinical rat model of HFpEF (male obese Zucker fatty spontaneously hypertensive heart failure F_1_ hybrid, ZSF1) following completion of either 8 weeks (6 days/week) of heat therapy (HEAT; 536 ± 18 g; *n* = 10) or control treatment (CON; 521 ± 23 g; *n* = 10). Obese ZSF1 rats develop multiple pathophysiological features that resemble clinical HFpEF, including preserved left ventricular ejection fraction, diastolic dysfunction, cardiac fibrosis, left atrial enlargement, systemic hypertension, pulmonary congestion, diabetes, skeletal muscle abnormalities and exercise intolerance​. These characteristics typically emerge between 10 and 20 weeks of age and make the model particularly suitable for evaluating interventions targeting both cardiac and peripheral impairments (Belbis et al., 2023; Espino‐Gonzalez et al., 2021; Nguyen et al., 2020; Schauer et al., 2020; Stolina et al., 2020).

### 2.3 Part A: Noradrenergic regulation of skeletal muscle ${P_{O_{2}}}_{\mathrm{is}}$ in health

#### 2.3.1 Surgical procedures

Rats were initially anaesthetized with a 3–5% isoflurane‐O_2_ mixture (Akron Animal Health; Lake Forest, IL, USA). Anaesthetized rats were kept on a heating pad to maintain core temperature at ∼37–38°C as measured via a rectal probe. Subsequently, while maintained on 1–2% isoflurane O_2_, the caudal (tail) artery was cannulated (PE‐10 connected to PE‐50; SAI Infusion Technologies; Lake Villa, IL, USA) for continuous monitoring of mean arterial pressure (MAP) and heart rate (HR) (ADInstruments; Sydney, Australia). The left spinotrapezius muscle was then exposed by carefully reflecting the overlying skin and fascia from the middorsal aspect of the rat. The exposed muscle was moistened frequently via superfusion of Krebs–Henseleit bicarbonate‐buffered solution (4.7 mM KCl, 2.0 mM CaCl_2_, 2.4 mM MgSO_4_, 131 mM NaCl, and 22 mM NaHCO_3_; pH 7.4; equilibrated with 5% CO_2_ and 95% N_2_, at ∼37–38°C) and the surrounding tissue covered with Saran wrap (SC Johnson; Racine, WI, USA). Stainless steel electrodes were sutured to the rostral (cathode) and caudal (anode) regions of the spinotrapezius for electrically induced contractions. Previous reports indicate that these surgical procedures do not impair the microvascular integrity or responsiveness of the spinotrapezius muscle (Bailey et al., 2000). Next, healthy Sprague–Dawley rats were administered dexmedetomidine hydrochloride (0.4 mg/kg i.p.; Dexdomitor; Zoetis Inc.; Parsippany‐Troy Hills, NJ, USA), and isoflurane inhalation was progressively reduced and eventually discontinued prior to experimental procedures (Belbis et al., 2024). Adequate depth of anaesthesia was confirmed and subsequently monitored via the absence of palpebral and toe pinch reflexes, and changes in heart rate and blood pressure. The current preparation retains vasomotor control such that muscle blood flow increases in the same proportion with O_2_ utilization as found in the exercising human (i.e., ∼5–6 L/min: 1 L/min) (Belbis et al., 2024; Ferreira, McDonough, et al., 2006; Hirai et al., 2018; Poole et al., 2011).

The Oxyphor probe G4 (Pd‐*meso*‐tetra‐(3,5‐dicarboxyphenyl)‐tetrabenzoporphyrin; 10 µM solution) was delivered directly to the tissue compartment of the left spinotrapezius muscle via the microinjection technique for ${P_{O_{2}}}_{\mathrm{is}}$ measurements (Hirai et al., 2018; Smith et al., 2002). Approximately four separate G4 microinjections (5–10 µL each) were performed with a 29‐gauge needle and a 1 mL syringe (Exelint International; Redondo Beach, CA, USA) along the length of the muscle dorsal aspect. A minimum of 15 min was allowed for uniform distribution of the injected probe within the muscle before any experimental procedure was initiated, as described below.

#### 2.3.2 Experimental protocol

The effects of topical NA administration (i.e., superfusion, 1 mL of a 5 × 10^−4 ^M solution, warmed to ∼37–38°C; Sigma‐Aldrich; St Louis, MO, USA) on spinotrapezius ${P_{O_{2}}}_{\mathrm{is}}$ were assessed at rest and during submaximal muscle contractions (0.5 Hz, ∼5 V, 2 ms pulse duration; Harvard Apparatus; Holliston, MA, USA). The NA superfusion concentration used herein was based on preliminary experiments demonstrating a reduction in spinotrapezius muscle ${P_{O_{2}}}_{\mathrm{is}}$ without eliciting elevations in MAP, consistent with local NA effects and absence of physiologically relevant leakage into the systemic circulation (cf. Ferreira, Padilla, et al., 2006).

**FIGURE 1:**
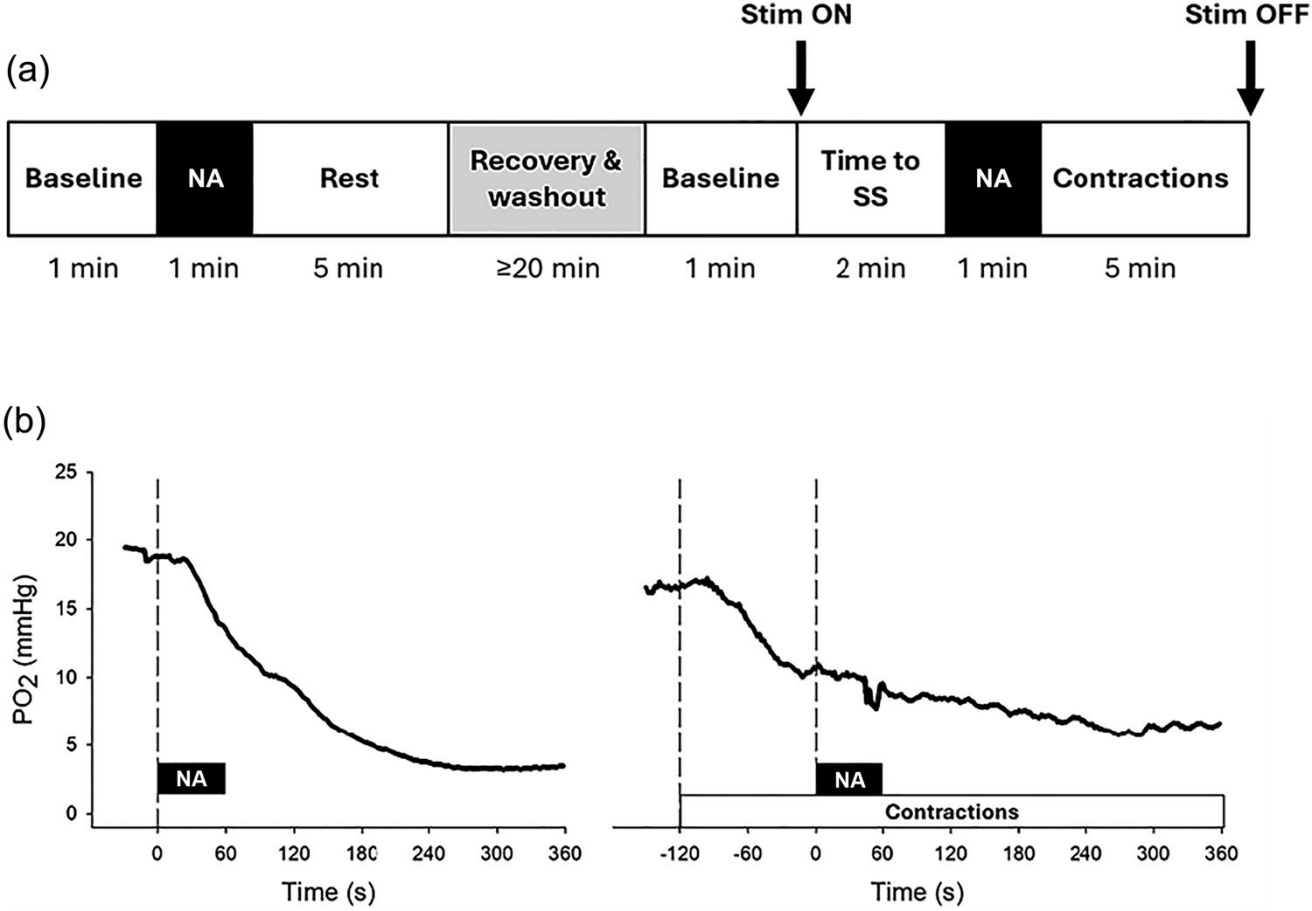
(a) Schematic representation of the experimental protocol (diagram not to scale). Baseline, pre‐NA ${P_{O_{2}}}_{\mathrm{is}}$; NA, noradrenaline superfusion; Rest, post‐NA ${P_{O_{2}}}_{\mathrm{is}}$ at rest; Stim ON, start of muscle contractions; Time to SS, 2 min of muscle contractions to achieve ${P_{O_{2}}}_{\mathrm{is}}$ steady‐state; Contractions, post‐NA ${P_{O_{2}}}_{\mathrm{is}}$ during muscle contractions; Stim OFF, cessation of muscle contractions. (b) Original recordings of spinotrapezius muscle ${P_{O_{2}}}_{\mathrm{is}}$ to NA at rest (left panel) and during contractions (right panel) in healthy rats.

The overall experimental design is illustrated in Figure 1. First, pre‐NA superfusion ${P_{O_{2}}}_{\mathrm{is}}$ (baseline) was measured for 1 min. NA was then superfused onto the spinotrapezius within a 1‐min period, and post‐NA ${P_{O_{2}}}_{\mathrm{is}}$ was recorded at rest for 5 min. A recovery period of ≥20 min was allowed while the muscle was superfused thoroughly with Krebs–Henseleit to wash out NA. Next, pre‐NA ${P_{O_{2}}}_{\mathrm{is}}$ (baseline) was measured for 1 min as described above for the resting condition. Submaximal muscle contractions were then evoked for 2 min (thus allowing ${P_{O_{2}}}_{\mathrm{is}}$ to reach steady‐state) (Hirai et al., 2018; Hirai, Craig, et al., 2019) before the 1‐min NA superfusion period. After post‐NA ${P_{O_{2}}}_{\mathrm{is}}$ was measured during contractions for 5 min, electrically induced muscle contractions were terminated. The stimulation protocol evokes an approximately four‐ to five‐fold increase in muscle blood flow together with approximately six‐ to seven‐fold increase in metabolic rate (which corresponds to ∼30% spinotrapezius ${\dot{V}}_{O_{2}\mathrm{peak}}$) above resting with either minor or no alterations in blood pH that are consistent with moderate intensity exercise (Behnke et al., 2001; Hirai et al., 2013). Our previous investigations demonstrate that the spinotrapezius preparation exhibits reproducible resting and contracting muscle blood flow, O_2_ utilization, and $P_{O_{2}}$ responses with no time‐related or ordering effects (Hirai et al., 2013).

#### 2.3.3 Skeletal muscle ${P_{O_{2}}}_{\mathrm{is}}$ measurement

Spinotrapezius muscle ${P_{O_{2}}}_{\mathrm{is}}$ was measured via phosphorescence quenching using a frequency domain phosphorometer (OxyLED TD, Oxygen Enterprises; Philadelphia, PA, USA) and the phosphorescent probe Oxyphor G4 (Esipova et al., 2011). Briefly, the Stern–Volmer relationship describes quantitatively the O_2_ dependence of the phosphorescent probe as follows:

$$P_{O_{2}}=\left[\left(\tau^{\circ }/\tau \right)-1\right]/\left(k_{Q}\times \tau^{\circ }\right)$$

where $k_{Q}$ is the quenching constant and $\tau^{\circ }$ and τ are the phosphorescence lifetimes in the absence of O_2_ and at a given $P_{O_{2}}$, respectively (Rumsey et al., 1988). G4‐specific values of $k_{Q}$ and $\tau^{\circ }$ in the physiological range (pH ∼7.4 and temperature of 38°C) are 304 mmHg^−1^ s^−1^ and 218 µs, respectively (Esipova et al., 2011). Phosphorescence lifetimes τ are independent of local probe concentration and insensitive to endogenous chromophores (Wilson et al., 2006; Yu et al., 2013). The common end of the bifurcated light guide was placed ∼2–4 mm superficial to the dorsal surface of the exposed spinotrapezius muscle. The phosphorometer modulates sinusoidal excitation frequencies between 100 Hz and 20 kHz, which allows phosphorescence lifetime measurements from 10 µs to ∼2.5 ms. The excitation light (635 nm wavelength; penetration depth of ∼500 µm) was focused on a randomly selected surface area of ∼2 mm diameter of exposed muscle devoid of large visible vessels to minimize the potential for macrovascular influences. ${P_{O_{2}}}_{\mathrm{is}}$ was recorded at 2 s intervals during experimental procedures. Following completion of ${P_{O_{2}}}_{\mathrm{is}}$ measurements, rats were euthanized via isoflurane overdose, followed by pneumothorax.

#### 2.3.4 Analysis of skeletal muscle ${P_{O_{2}}}_{\mathrm{is}}$

The noradrenergic response was evaluated based on the noradrenaline‐induced changes in ${P_{O_{2}}}_{\mathrm{is}}$ at rest and during the contraction steady‐state using (1) the absolute and relative ${P_{O_{2}}}_{\mathrm{is}}$ decrease with noradrenaline normalized to MAP (Δ${P_{O_{2}}}_{\mathrm{is}}$/MAP), and (2) the area under the ${P_{O_{2}}}_{\mathrm{is}}$–time curve normalized to MAP (AUC/MAP). Similar to previous studies (Chavoshan et al., 2002; Fadel et al., 2004; Hansen et al., 1996; Sprick et al., 2019; Teixeira et al., 2022; Vongpatanasin et al., 2011), the magnitude of functional sympatholysis was determined as the difference between noradrenaline‐induced changes in skeletal muscle oxygenation at rest and during the contraction steady‐state (i.e., absolute and relative Δ${P_{O_{2}}}_{\mathrm{is}}$/MAP_(contractions–rest)_ and AUC/MAP_(contractions–rest)_).

### 2.4 Part B: Noradrenergic regulation of skeletal muscle ${P_{O_{2}}}_{\mathrm{is}}$ in HFpEF: Impact of heat therapy

#### 2.4.1 Rat model of heart failure with preserved ejection fraction

Obese ZSF1 rats were acquired at 8–10 weeks of age and aged in‐house until at least 15 weeks before undergoing experimental procedures. Once they reached ∼15–20 weeks of age, pre‐intervention measurements were obtained as described below, and control or heat therapy interventions were subsequently initiated. As mentioned above, this animal model develops signs and symptoms consistent with HFpEF between ∼10 and 20 weeks of age (Belbis et al., 2025; Espino‐Gonzalez et al., 2021; Leite et al., 2015; Nguyen et al., 2020). Animals were then randomized to undergo 8 weeks of HEAT (*n* = 10) or CON (*n* = 10) interventions. Body weight and food consumption were assessed daily throughout the intervention period. Post‐intervention outcomes were evaluated at least 24 h after the final treatment session.

#### 2.4.2 Core body temperature and locomotor activity monitoring

Data loggers were implanted intraperitoneally to measure core temperature and accelerometer‐derived activity (external acceleration; AvgEA) throughout the study (DST micro‐ACT; Star‐Oddi Ltd, Gardabaer, Iceland). Under 3–5% isoflurane–O_2_ mixture anaesthesia, an incision was made in the linea alba, and the data logger was carefully inserted into the abdominal cavity without fixation to the muscular layer of the abdominal wall. Subsequently, the muscular layer and the outer skin were sutured separately, and carprofen (5 mg/kg; Rimadyl, Zoetis Inc.) administered subcutaneously for postoperative analgesia. The surgical site was monitored daily for signs of infection or complications throughout the study. Animals were given at least 5 days to recover from surgeries before undergoing subsequent procedures. Upon completion of the study and euthanasia, data loggers were recovered, and information was extracted using the manufacturer's communication box and software (Mercury 6.69; Star‐Oddi Ltd.).

Temperature and activity data were collected using two alternating measurement intervals: a 4‐min interval during which temperature was recorded for 1 min, followed by a 3‐min interval during which both temperature and activity were recorded for 1 min. These intervals alternated continuously throughout the recording period. As a result, temperature was measured every 3–4 min (approximately 0.004–0.0056 Hz), while activity was measured every 7 min (approximately 0.0024 Hz) over the course of the 8‐week intervention. Due to technical constraints, only data from Weeks 2, 4, and 6 are reported herein for HFpEF‐CON (*n* = 10); HFpEF‐HEAT (*n* = 10).

#### 2.4.3 Cardiac function and morphology

The echocardiography techniques used herein have been described in detail previously (Dann et al., 2022). Briefly, rats were anaesthetized initially with a 3–5% isoflurane–O_2_ mixture, and then maintained on 1–2.5% isoflurane–O_2_ and placed on a heating pad to keep core temperature at ∼37–38°C as measured via a rectal probe. Echocardiographic images were acquired using a Vevo 3100 high‐frequency small animal ultrasound system (FUJIFILM VisualSonics; Toronto, Canada) with the MX250 linear array transducer with centre transmit frequency of 21 MHz, 15–30 MHz bandwidth and axial resolution of 75 µm. Images of the left ventricle (LV) were taken at pre‐ and post‐intervention using parasternal long‐axis (PLAX) and short‐axis (SAX) views in two‐dimensional (2D) brightness mode (B‐mode). 2D motion mode (M‐mode) images of the heart were also obtained in PLAX and SAX views.

PLAX and SAX B‐mode and M‐mode images were analysed on Vevo LAB (FUJIFILM VisualSonics; Toronto, Canada). Automated tracings, using the AutoLV function, were completed to assess LV function and structure pre‐ and post‐intervention. Additionally, manual LV contours were completed in the PLAX B‐mode images by tracing the endocardium during both end‐systole and end‐diastole using Vevo LAB's operator‐defined LV trace function. The following parameters were assessed: ejection fraction (EF), left ventricular end‐systolic volume (LVESV), LV end‐diastolic volume (LVEDV), stroke volume (SV), cardiac output (CO), LV end‐systolic diameter (LVIDs), LV end‐diastolic diameter (LVIDd), fractional shortening (FS), LV anterior wall thickness in systole (LVAWs), LV anterior wall thickness in diastole (LVAWd), LV posterior wall thickness in systole (LVPWs), LV posterior wall thickness in diastole (LVPWd), *E*/*A* ratio, and isovolumic relaxation time (IVRT).

#### 2.4.4 Body composition

Whole‐body lean and fat mass were assessed via magnetic resonance imaging (EchoMRI 500 analyser; EchoMRI LLC; Houston, TX, USA) pre‐intervention (Week 0) and post‐intervention (Week 8). Measurements were performed in awake, unanaesthetized animals. Two consecutive scans were taken for each animal at each time point, and the average of the two trials was reported.

#### 2.4.5 Heat therapy

HFpEF rats were subjected to either ∼8 weeks (6 days/week) of HEAT or CON interventions. Animals in the HEAT group were placed into a powered‐on incubator (Heratherm Refrigerated Incubators; Thermo Fisher Scientific; Waltham, MA, USA) set to 39°C, whereas those in the CON group were placed into powered‐off incubators or cabinets maintained at room temperature (∼22°C). The selection of 39°C as the treatment temperature was based on previous studies from our group that demonstrated its efficacy and safety in mouse models of obesity and ischaemia‐induced muscle injury. Specifically, repeated exposure to HT at 39°C attenuated diet‐induced fat accumulation and rescued skeletal muscle contractile dysfunction (Kim et al., 2019, 2021).

**FIGURE 2:**
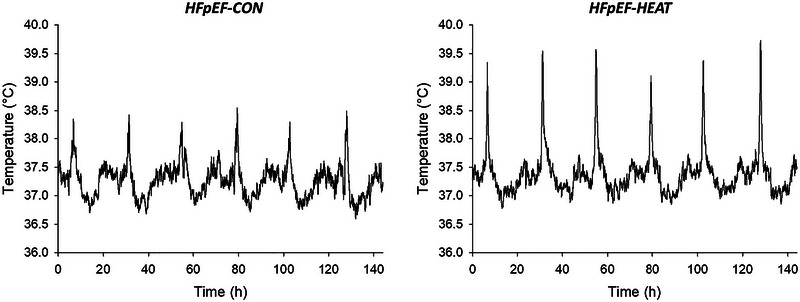
Mean core temperatures over the course of a week of treatment (Week 6) in HFpEF‐CON (*n* = 10) and HFpEF‐HEAT (*n* = 10) groups. Error bars omitted for clarity.

All animals remained in individual cages without food or water during treatment sessions. Exposure duration increased progressively from 5 to 20 min daily over the initial 7 weeks to allow acclimation and minimize the risk of heat stress. Total weekly treatment durations were as follows: Week 1, 50 min; Week 2, 80 min; Week 3, 95 min; Week 4, 105 min; Week 5, 107.5 min; Week 6, 112.5 min; Week 7, 117.5 min; and Week 8, 120 min (Belbis et al., 2025). As mentioned above, our previous studies indicate that this heating protocol is safe and effective in eliciting multiple central and peripheral adaptations that improve exercise tolerance in HFpEF rats (Belbis et al., 2025). Mean core temperatures over the course of a week of intervention in HFpEF‐CON and HFpEF‐HEAT herein are depicted in Figure 2.

#### 2.4.6 Surgical procedures, experimental protocol, skeletal muscle ${P_{O_{2}}}_{\mathrm{is}}$ measurement and analysis

All surgical and experimental procedures were identical to those described above for healthy Sprague–Dawley rats (Part A), except that obese ZSF1 (HFpEF) rats were maintained under 1–2% isoflurane in 21% O_2_ anaesthesia (as opposed to intraperitoneal dexmedetomidine hydrochloride) following catheter placement and spinotrapezius exposure. This approach minimizes the potential influence of dexmedetomidine on sympathetic regulation via its α₂‐adrenergic agonist properties (Kenney et al., 2014). The level of anaesthesia was monitored frequently via the palpebral and toe‐pinch reflexes, and changes in heart rate and blood pressure were supplemented as necessary. Spinotrapezius ${P_{O_{2}}}_{\mathrm{is}}$ measurement and data analysis were performed as described above for healthy rats.

#### 2.4.7 Tissue sampling

At the end of the experimental protocol, rats were euthanized as described above for healthy rats (i.e., isoflurane overdose followed by pneumothorax). The spinotrapezius (mixed fibre type muscle), soleus (predominantly slow‐twitch, type I fibre muscle), extensor digitorum longus (EDL, predominantly fast‐twitch, type II fibre muscle) (Delp & Duan, 1996; Leek et al., 2001), lungs and heart were dissected, cleared of visible fat and connective tissue and weighed. Tissue sampling was performed on four HFpEF‐CON and 10 HFpEF‐HEAT rats. The smaller number in the control group reflects the inclusion of animals from preliminary work in which tissue weights were not determined.

### 2.5 Statistical analysis

Data were analysed using Student's unpaired *t*‐test, one‐way ANOVA, two‐way repeated measures mixed‐effects ANOVA, or linear mixed‐effects models, as appropriate. Normality was assessed using the Shapiro–Wilk test, and homogeneity of variances using Levene's test. Welch's tests were applied when variances were unequal. *Post hoc* comparisons were corrected for multiple comparisons using the Bonferroni method, Tukey's HSD, or the Games–Howell test, as appropriate. Analyses were performed using Jamovi (version 2.6.26; The Jamovi Project, Sydney, Australia). Data are presented as means ± SD. Statistical significance was set at *P* <  0.05.

## 3 RESULTS

### 3.1 Part A: Noradrenergic regulation of skeletal muscle ${P_{O_{2}}}_{\mathrm{is}}$ in health

MAP and HR at rest and during muscle contractions prior to and after noradrenaline superfusion are shown in Table 1. As expected based on the topical drug delivery method, no changes in either MAP or HR were observed with local noradrenaline at rest or during muscle contractions in healthy rats (*P *> 0.05 for all).

**TABLE 1:** Mean arterial pressure (MAP) and heart rate (HR) at rest and during muscle contractions prior to and after noradrenaline superfusion in healthy, HFpEF‐CON and HFpEF‐HEAT anaesthetized rats.

	Healthy	HFpEF‐CON	HFpEF‐HEAT
MAP (mmHg)			
Rest, pre‐NA	130 ± 26^*^	96 ± 3	94 ± 4
Rest, post‐NA	135 ± 29^*^	101 ± 6	103 ± 3
Contractions, pre‐NA	135 ± 22^*^	98 ± 6	99 ± 6
Contractions, post‐NA	145 ± 19^*^	101 ± 7	106 ± 7
HR (bpm)			
Rest, pre‐NA	238 ± 20^*^	282 ± 14	295 ± 21
Rest, post‐NA	238 ± 23^*^	283 ± 14	296 ± 18
Contractions, pre‐NA	240 ± 31^*^	275 ± 17	280 ± 17
Contractions, post‐NA	252 ± 25^*^	280 ± 20	277 ± 16

*Note*: Values are means ± SD. Healthy, *n* = 14; HFpEF‐CON, *n* = 10; HFpEF‐HEAT, *n* = 10. ^*^Significantly different from HFpEF‐CON and HFpEF‐HEAT. Abbreviations: HFpEF, heart failure with preserved ejection fraction; NA, noradrenaline.

**FIGURE 3:**
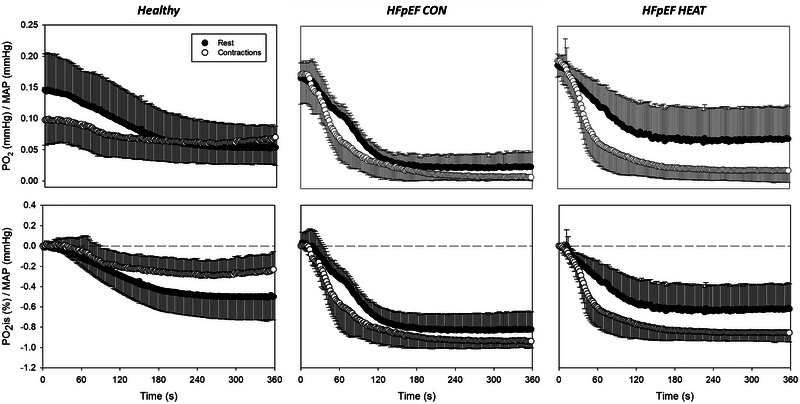
Absolute (top panels) and relative (bottom panels) muscle ${P_{O_{2}}}_{\mathrm{is}}$ responses normalized to mean arterial pressure (${P_{O_{2}}}_{\mathrm{is}}$/MAP) with NA superfusion at rest and during contractions. Values are means ± SD. Healthy, *n* = 14; HFpEF‐CON, *n* = 10; and HFpEF‐HEAT, *n* = 10. Time zero denotes the onset of noradrenaline superfusion.

**FIGURE 4:**
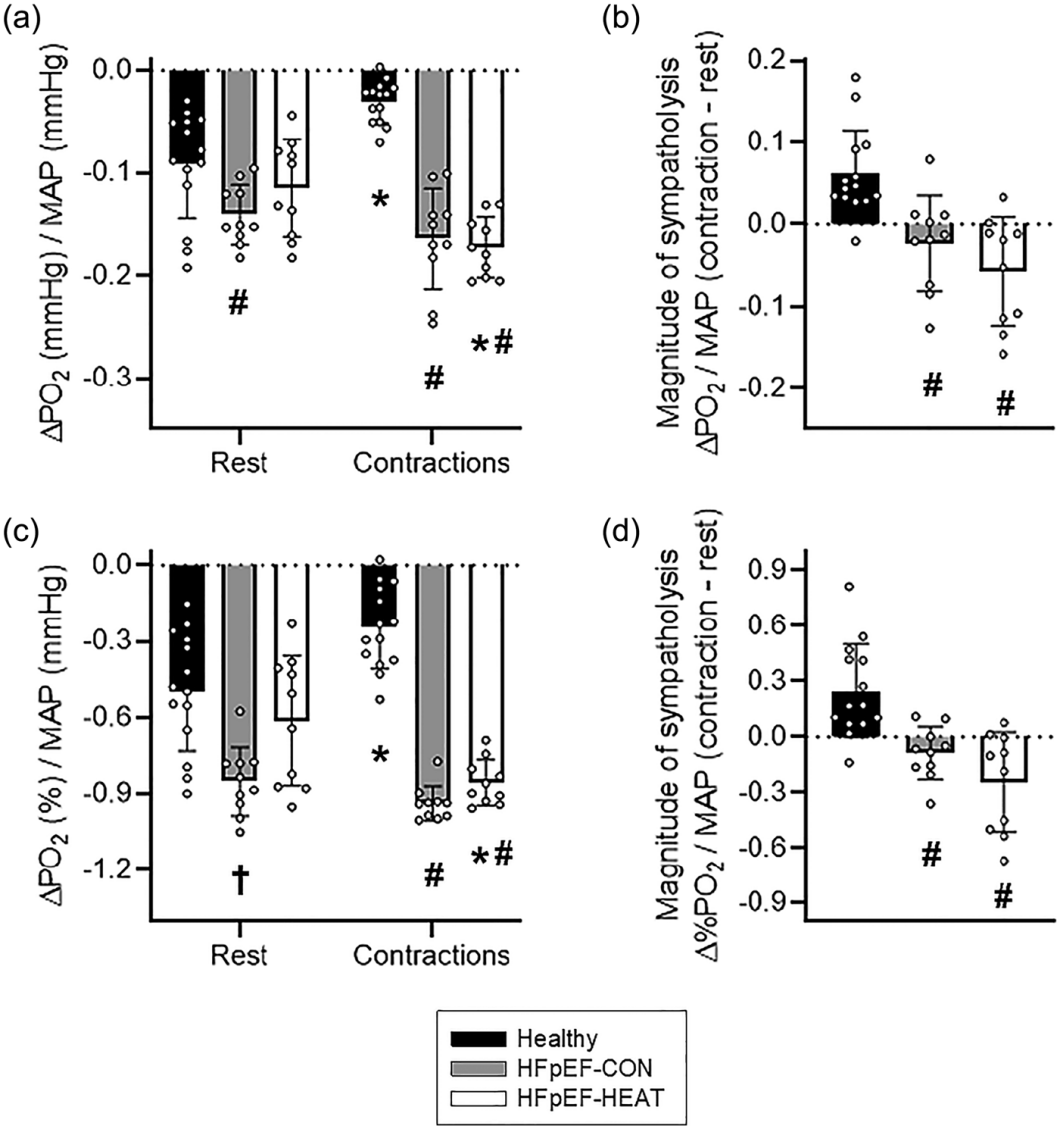
(a) Individual and mean absolute change in muscle ${P_{O_{2}}}_{\mathrm{is}}$ with NA normalized to mean arterial pressure (Δ$P_{O_{2}}$/MAP) at rest and during contractions. 95% confidence intervals: Healthy rest, −0.12, −0.06; Healthy contractions, −0.04, −0.02; HFpEF‐CON rest, −0.16, −0.12; HFpEF‐CON contractions, −0.20, −0.13; HFpEF‐HEAT rest, −0.15, −0.08; HFpEF‐HEAT contractions, −0.19, −0.15. (b) Magnitude of sympatholysis as the difference in absolute Δ$P_{O_{2}}$/MAP to noradrenaline between rest and contractions. 95% confidence intervals: Healthy, 0.03, 0.09; HFpEF‐CON, −0.07, 0.02; HFpEF‐HEAT, −0.10, −0.01. (c) Relative Δ$P_{O_{2}}$/MAP at rest and during contractions. 95% confidence intervals: Healthy rest, −0.63, −0.36; Healthy contractions, −0.34, −0.15; HFpEF‐CON rest, −0.95, −0.76; HFpEF‐CON contractions, −0.99, −0.89; HFpEF‐HEAT rest, −0.79, −0.43; HFpEF‐HEAT contractions, −0.92, −0.79. (d) Magnitude of sympatholysis as the difference in relative Δ$P_{O_{2}}$/MAP to noradrenaline between rest and contractions. 95% confidence intervals: Healthy, 0.10, 0.40; HFpEF‐CON, −0.19, 0.02; HFpEF‐HEAT, −0.43, −0.05. Healthy, *n* = 14; HFpEF‐CON, *n* = 10; HFpEF‐HEAT, *n* = 10. Values are means ± SD. Significantly different from: *rest, #healthy, †healthy and HFpEF‐HEAT.

**FIGURE 5:**
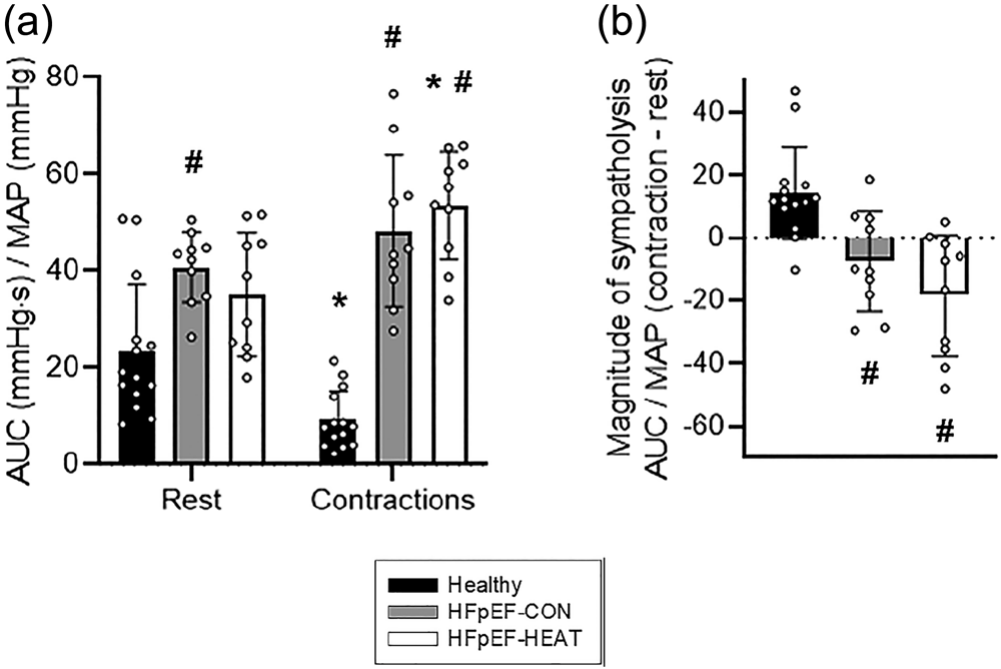
(a) Individual and mean values of the area under the muscle ${P_{O_{2}}}_{\mathrm{is}}$ curve following NA superfusion normalized to mean arterial pressure (AUC/MAP) at rest and during contractions. 95% confidence intervals: Healthy Rest, 15.4, 31.5; Healthy contractions, 5.8, 12.7; HFpEF‐CON rest, 35.5, 46.0; HFpEF‐CON contractions, 37.1, 59.5; HFpEF‐HEAT rest, 26.0, 44.3; HFpEF‐HEAT contractions, 45.5, 61.4. (b) Magnitude of sympatholysis as the difference between the AUC/MAP in response to noradrenaline at rest and during muscle contractions. Healthy, 5.7, 22.8; HFpEF‐CON, −18.9, 3.9; HFpEF‐HEAT, −32.1, −4.5. Healthy, *n* = 14; HFpEF‐CON, *n* = 10; HFpEF‐HEAT, *n* = 10. Values are means ± SD. Significantly different from: *rest, #healthy.

Mean temporal muscle ${P_{O_{2}}}_{\mathrm{is}}$/MAP responses to noradrenaline superfusion at rest and during contractions are presented in Figure 3. Local noradrenaline lowered muscle ${P_{O_{2}}}_{\mathrm{is}}$/MAP at rest (*P *< 0.05), but not during exercise (*P *> 0.05; Table 2). Thus, consistent with functional sympatholysis, muscle contractions attenuated the noradrenaline‐evoked reductions in spinotrapezius interstitial oxygenation as evaluated by the Δ${P_{O_{2}}}_{\mathrm{is}}$/MAP and AUC/MAP in 13 out of 14 healthy rats (*P *< 0.05 for both; Figures 4 and 5).

**TABLE 2:** Absolute muscle ${P_{O_{2}}}_{\mathrm{is}}$ (mmHg) normalized to MAP (mmHg) at rest and during contractions pre‐ and post‐NA superfusion in healthy, HFpEF‐CON and HFpEF‐HEAT rats.

	Healthy		HFpEF‐CON		HFpEF‐HEAT	
	Pre‐NA	Post‐NA	Pre‐NA	Post‐NA	Pre‐NA	Post‐NA
Rest	0.15 ± 0.06	0.05 ± 0.03^*^	0.17 ± 0.03	0.02 ± 0.02^*^	0.18 ± 0.02	0.07 ± 0.05^*^
Contractions	0.10 ± 0.04^†^	0.07 ± 0.04	0.17 ± 0.05^#^	0.01 ± 0.01^*#^	0.19 ± 0.03^#^	0.02 ± 0.02^*†^

*Note*: Values are means ± SD. Healthy, *n* = 14; HFpEF‐CON, *n* = 10; HFpEF‐HEAT, *n* = 10. Significantly different from: ^*^pre‐NA within group; ^#^healthy within condition; ^†^rest within condition. Abbreviations: HFpEF, heart failure with preserved ejection fraction; NA, noradrenaline.

### 3.2 Part B: Noradrenergic regulation of skeletal muscle ${P_{O_{2}}}_{\mathrm{is}}$ in HFpEF: Impact of heat therapy

**FIGURE 6:**
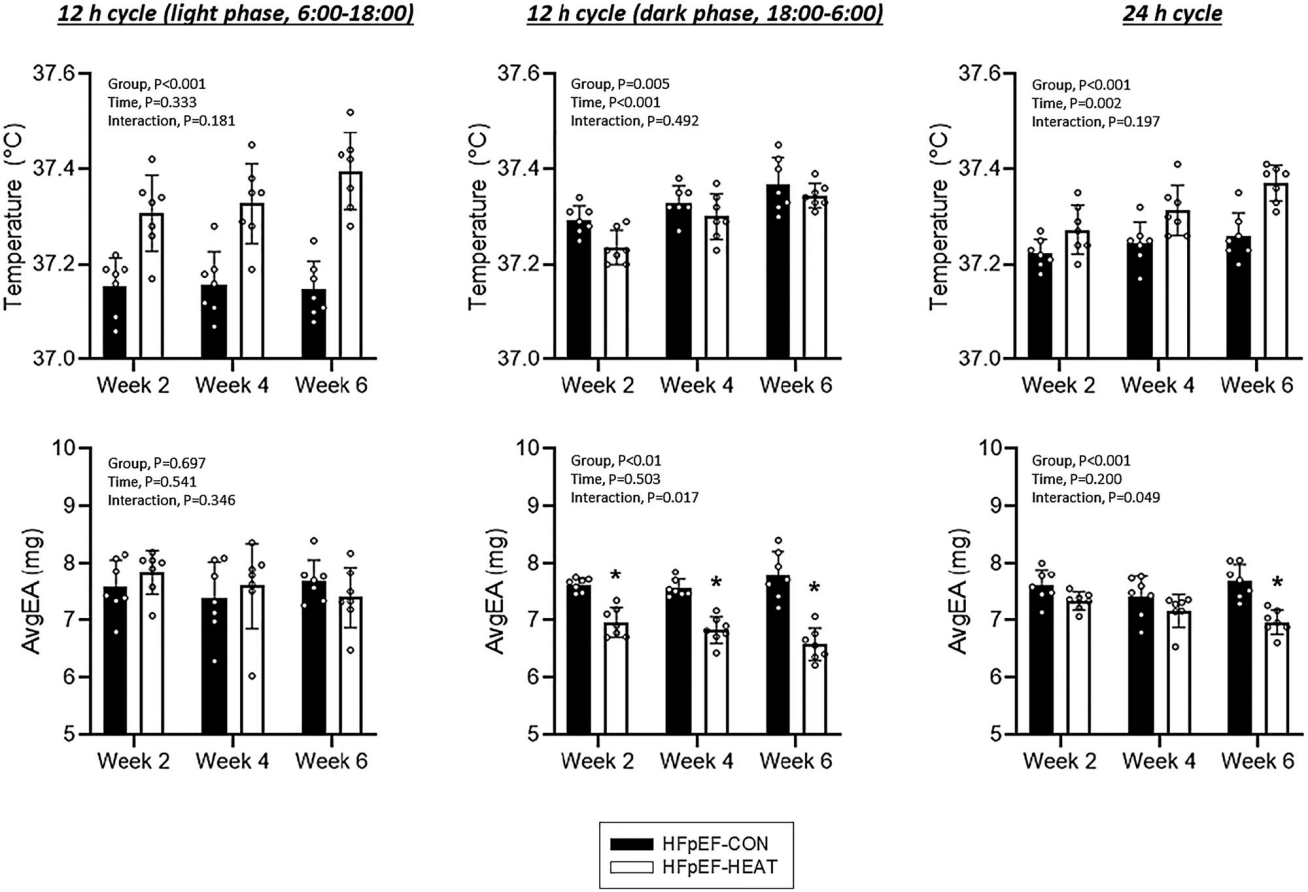
Top panels: individual and mean core temperatures across different light/dark cycles in HFpEF‐CON and HFpEF‐HEAT groups. Bottom panels: individual and mean external acceleration (AvgEA; index of physical activity) across different light/dark cycles in HFpEF‐CON and HFpEF‐HEAT groups. Data are shown during the light phase (12 h cycle: 06.00–18.00 h; left panels), dark phase (12 h cycle: 18.00–06.00 h; middle panels), or continuously over 24 h (right panels) at Weeks 2, 4 and 6. Values are means ± SD. HFpEF‐CON, *n* = 10; HFpEF‐HEAT, *n* = 10. *Significantly different from HFpEF‐CON.

Core temperatures and physical activity (AvgEA) across different light/dark cycles in HFpEF‐CON and HFpEF‐HEAT are shown in Figure 6. During the 12 h light phase (06.00–18.00 h) and 24 h cycles, HFpEF‐HEAT exhibited higher core temperatures than HFpEF‐CON (*P *< 0.001), whereas in the 12 h dark phase (18.00–06.00 h) cycle, core temperature was lower in the HFpEF‐HEAT group (*P* = 0.005). The significant time effects observed in the 24 h (*P* = 0.002) cycle reflect the progressive heating protocol used herein. AvgEA did not differ between groups in the 12 h light‐phase cycle. However, in the 12 h dark phase, HFpEF‐HEAT animals showed reduced AvgEA at Weeks 2, 4 and 6 (*P *< 0.05). Light–dark AvgEA did not differ in HFpEF‐CON (*P *> 0.05), but was lower during the dark phase in HFpEF‐HEAT (*P *< 0.05). In the 24 h cycle, avgEA was lower at Week 6 in the HFpEF‐HEAT group (*P *< 0.05).

Body mass and composition in HFpEF‐CON and HFpEF‐HEAT are shown in Table 3. While both groups experienced an increase in total body mass from pre‐ to post‐intervention (*P *< 0.05 for both), HFpEF‐HEAT had greater total body mass at post‐intervention than HFpEF‐CON (*P *< 0.05). Moreover, no differences in body composition (i.e., absolute or relative lean mass or fat mass) were observed between groups (*P *> 0.05 for all). Similarly, there were no differences in tissue weights for the spinotrapezius, soleus, EDL, lung, or heart between groups (*P *> 0.05 for all; Table 4).

**FIGURE 7:**
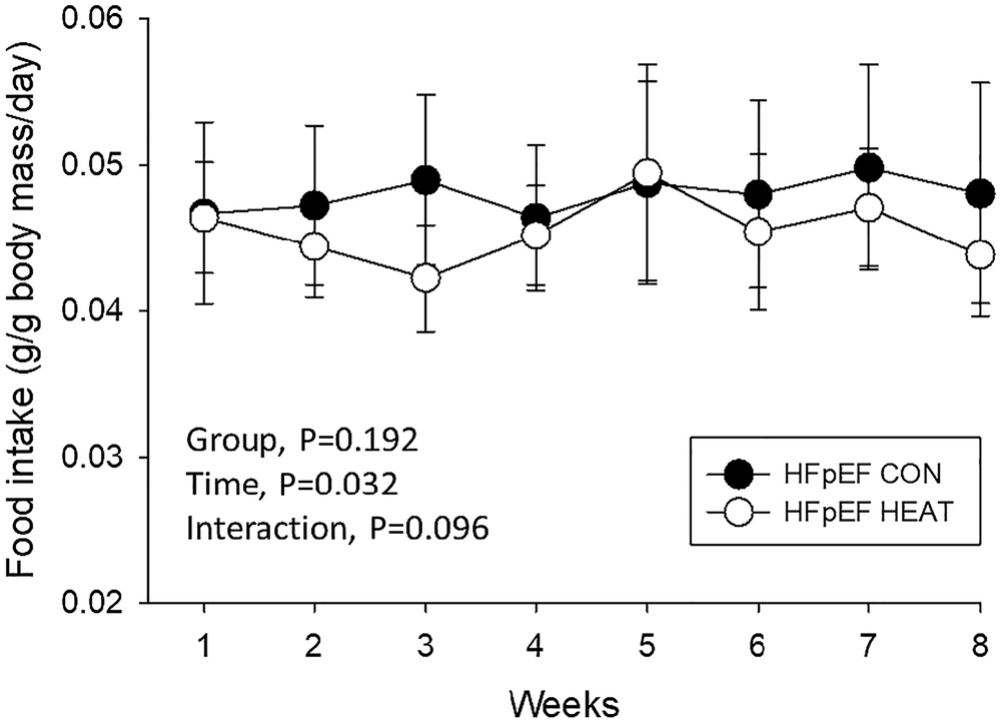
Mean food intake normalized to body mass across 8 weeks in HFpEF‐CON (*n* = 10) and HFpEF‐HEAT (*n* = 10) groups. Values are means ± SD.

Mean food intake normalized to body mass during the 8‐week intervention period is illustrated in Figure 7. There were no differences in food intake between HFpEF‐CON and HFpEF‐HEAT throughout the study (*P *> 0.05), although a main effect of time was observed (*P* = 0.032).

Doppler echocardiography measurements made pre‐ and post‐intervention in HFpEF‐CON and HFpEF‐HEAT rats are presented in Table 5. There were no differences in cardiac structure or function between groups (*P *> 0.05 for all), except for the increased IVRT from pre‐ to post‐intervention in HFpEF‐HEAT (*P *< 0.05).

**TABLE 3:** Body mass and composition pre‐ and post‐intervention in HFpEF‐CON and HFpEF‐HEAT rats.

	HFpEF‐CON		HFpEF‐HEAT	
	Pre	Post	Pre	Post
Total body mass (g)	521 ± 23	583 ± 27^*^	536 ± 18	628 ± 20^*†^
Lean mass (g)	297 ± 18	292 ± 44	301 ± 16	320 ± 14
Lean mass/total mass (g/g)	0.57 ± 0.01	0.50 ± 0.07	0.56 ± 0.02	0.51 ± 0.02
Fat mass (g)	196 ± 10	187 ± 16	211 ± 9	212 ± 28
Fat mass/total mass (g/g)	0.38 ± 0.02	0.32 ± 0.02	0.39 ± 0.02	0.34 ± 0.04

*Note*: Values are means ± SD. HFpEF‐CON, *n* = 10; HFpEF‐HEAT, *n* = 10. Significantly different from: ^*^Pre within group; ^†^HFpEF‐CON within condition. Abbreviation: HFpEF, heart failure with preserved ejection fraction.

As expected, no changes in either MAP or HR were observed with local noradrenaline at rest or during contractions in HFpEF‐CON or HFpEF‐HEAT (*P *> 0.05; Table 1). The differences in MAP and HR between healthy and HFpEF groups can be ascribed to the anaesthetics used as mentioned above (i.e., isoflurane and dexmedetomidine hydrochloride, respectively; *P *< 0.05).

As displayed in Figure 3 and Table 2, noradrenaline superfusion lowered muscle ${P_{O_{2}}}_{\mathrm{is}}$/MAP both at rest and during contractions in HFpEF‐CON and HFpEF‐HEAT (*P *< 0.05 for all). While HFpEF‐CON had an exacerbated noradrenergic response at rest compared to healthy rats (i.e., both Δ${P_{O_{2}}}_{\mathrm{is}}$/MAP and AUC/MAP; *P *< 0.05), heat therapy in HFpEF restored those resting responses (*P *> 0.05 for HFpEF‐HEAT vs. healthy; Figures 4 and 5). Furthermore, ${P_{O_{2}}}_{\mathrm{is}}$ changes with noradrenaline during contractions were not different between HFpEF‐CON and HFpEF‐HEAT (*P *> 0.05). Consistent with our hypothesis, functional sympatholysis was impaired in HFpEF‐CON as evaluated by the Δ${P_{O_{2}}}_{\mathrm{is}}$/MAP and AUC/MAP (*P *< 0.05 for all). However, contrary to our hypothesis, heat therapy did not improve functional sympatholysis in HFpEF (Figures 4 and 5).

## 4 DISCUSSION

This investigation is the first to (1) examine noradrenergic regulation of skeletal muscle ${P_{O_{2}}}_{\mathrm{is}}$ in healthy and HFpEF rats, and (2) evaluate whether heat therapy could improve functional sympatholysis in HFpEF. The principal findings are as follows. Part A: as expected, muscle contractions in healthy rats blunted the noradrenaline‐induced reduction in ${P_{O_{2}}}_{\mathrm{is}}$ observed at rest, consistent with intact functional sympatholysis. Part B: HFpEF rats exhibited an exaggerated noradrenergic response at rest compared to healthy controls and, consistent with our hypothesis, functional sympatholysis was impaired. Heat therapy mitigated the exaggerated noradrenergic response at rest in HFpEF rats, such that ${P_{O_{2}}}_{\mathrm{is}}$ levels were no longer different from healthy controls. However, heat therapy had no effect on the ${P_{O_{2}}}_{\mathrm{is}}$ response to noradrenaline during contractions, with HFpEF‐HEAT animals showing no difference from HFpEF‐CON, indicating persistent impairment in functional sympatholysis. Finally, our results demonstrate that the combination of phosphorescence quenching (to assess muscle ${P_{O_{2}}}_{\mathrm{is}}$) and superfusion techniques can be effectively used to evaluate disease and treatment effects on functional sympatholysis in the anaesthetized rat.

### 4.1 Part A: Noradrenergic regulation of skeletal muscle ${P_{O_{2}}}_{\mathrm{is}}$ in health

Tissue ${P_{O_{2}}}_{\mathrm{is}}$ measurements via phosphorescence quenching were performed herein using the rat spinotrapezius muscle preparation (Bailey et al., 2000; Gray, 1973). As mentioned above, the rat spinotrapezius exhibits a mixed fibre‐type composition and oxidative capacity similar to those of the human quadriceps (Delp & Duan, 1996; Leek et al., 2001), thus representing a useful analogue of the human locomotor muscle. Due to its superficial anatomical location, the spinotrapezius can be surgically exposed with minimal disruption to neural or microvascular regulation (Bailey et al., 2000). Accordingly, the current anaesthetized preparation retains vasomotor control such that contracting muscle blood flow increases in proportion to metabolic rate as seen in the exercising human (Belbis et al., 2024; Ferreira, McDonough, et al., 2006; Hirai et al., 2018; Poole et al., 2011). Importantly, utilization of superfusion techniques with the small muscle mass spinotrapezius allows for (1) local drug administration, thereby minimizing alterations in central haemodynamics (e.g., MAP or HR), as demonstrated previously (Hirai et al., 2014, 2021) and confirmed herein (Table 1), and (2) direct evaluation of local oxygen delivery–utilization matching independent of any cardiopulmonary limitations imposed by the disease.

That muscle contractions blunted the decrease in spinotrapezius ${P_{O_{2}}}_{\mathrm{is}}$ with noradrenaline compared to the resting condition (Table 2, Figures 3, 4, 5) is consistent with functional sympatholysis. These responses are qualitatively similar to those reported in both humans and animal models using NIRS following a variety of sympathoexcitatory stimuli, including lower‐body negative pressure and lumbar nerve stimulation (Chavoshan et al., 2002; Fadel et al., 2004; Hansen et al., 1996; Sprick et al., 2019; Teixeira et al., 2022; Vongpatanasin et al., 2011). Pertinent to the present investigation, Fadel et al. (2004) demonstrated in the anaesthetized rat that electrical stimulation of sympathetic nerves produces graded decreases in both NIRS‐derived muscle oxygenation and blood flow in the resting hindlimb. During electrically induced muscle contractions, decreases in muscle oxygenation and blood flow elicited by sympathetic stimulation were attenuated. In that study, similar NIRS‐derived muscle oxygenation and blood flow responses were also found in humans with the use of lower‐body negative pressure at rest and during exercise (Fadel et al., 2004). The current phosphorescence quenching (${P_{O_{2}}}_{\mathrm{is}}$) data thus align with the previous NIRS responses to sympathetic activation in both humans and animals, supporting that changes in tissue oxygenation may be used to evaluate sympathetic vasoconstriction in skeletal muscle.

### 4.2 Part B: Noradrenergic regulation of skeletal muscle ${P_{O_{2}}}_{\mathrm{is}}$ in HFpEF: Impact of heat therapy

The effects of heat therapy on core temperature and physical activity (AvgEA) across different light–dark cycles in HFpEF rats are illustrated in Figure 6. As expected, HFpEF‐HEAT demonstrated higher core temperatures than HFpEF‐CON during the 12 h light phase, consistent with the time of day the animals received the interventions. Interestingly, the opposite was observed during the 12 h dark phase, with HFpEF‐HEAT exhibiting lower core temperatures than HFpEF‐CON. Nonetheless, HFpEF‐HEAT had progressively higher core temperatures than HFpEF‐CON during 24 h cycles, thereby reflecting the incremental heating protocol employed herein. While no group differences in AvgEA during the 12 h light cycle were detected, HFpEF‐HEAT had lower physical activity during the 12 h dark cycle and 24 h cycle. Lowered physical activity and thus core temperature in HFpEF‐HEAT during the 12 h dark cycle might represent a behavioural adaptation to minimize internal heat production and maintain thermal homeostasis (Raun et al., 2020). Regardless of the mechanism, reduced physical activity could explain, at least in part, the greater total body mass at post‐intervention in HFpEF‐HEAT compared to HFpEF‐CON (Table 3), considering that there were no group differences in food intake (Figure 7). It is interesting to note that heat therapy had no effects on body composition (i.e., fat mass or lean mass; Table 3) or skeletal muscle mass in HFpEF rats (i.e., spinotrapezius, soleus or EDL; Table 4). This is generally inconsistent with our prior reports of attenuated fat accumulation and enhanced skeletal muscle mass with heat therapy in preclinical models of peripheral artery disease (Kim et al., 2019, 2021) and HFpEF (Belbis et al., 2025). The resolution of this conflict is beyond the scope of this study. Furthermore, we found no difference in light–dark AvgEA in HFpEF‐CON animals, consistent with previous reports of blunted circadian rhythm amplitudes of activity in obese rats (Maskrey et al., 2001; Murakami et al., 1995) and reduced nocturnal activity in HFpEF rodents (Jacobsen et al., 2024).

**TABLE 4:** Absolute and relative tissue weights in HFpEF‐CON and HFpEF‐HEAT rats.

	HFpEF‐CON	HFpEF‐HEAT
Spinotrapezius (mg)	440 ± 57	470 ± 59
Spinotrapezius/body mass (mg/g)	0.74 ± 0.11	0.75 ± 0.10
Soleus (mg)	169 ± 24	178 ± 11
Soleus/body mass (mg/g)	0.28 ± 0.02	0.28 ± 0.02
EDL (mg)	181 ± 54	150 ± 21
EDL/body mass (mg/g)	0.30 ± 0.08	0.24 ± 0.03
Lung (mg)	1595 ± 230	1463 ± 104
Lung/body mass (mg/g)	2.71 ± 0.54	2.33 ± 0.20
Heart (mg)	1597 ± 253	1522 ± 126
Heart/body mass (mg/g)	2.68 ± 0.35	2.42 ± 0.17

*Note*: Values are means ± SD. HFpEF‐CON, *n* = 4; HFpEF‐HEAT, *n* = 10. Abbreviations: EDL, extensor digitorum longus; HFpEF, heart failure with preserved ejection fraction.

**TABLE 5:** Doppler echocardiographic data assessment of left ventricle structure and function pre‐ and post‐intervention in HFpEF‐CON and HFpEF‐HEAT rats.

	HFpEF‐CON		HFpEF‐HEAT	
	Pre	Post	Pre	Post
SV (µL)	332.4 ± 84.5	348.0 ± 47.4	333.7 ± 53.2	314.1 ± 56.9
CO (ml/min)	96.0 ± 25.5	101.6 ± 12.0	99.1 ± 15.8	95.1 ± 20.2
EF (%)	51.3 ± 4.2	51.6 ± 2.3	51.0 ± 2.6	52.5 ± 3.8
LVIDd (mm)	8.2 ± 0.5	8.6 ± 0.6	8.6 ± 0.5	8.3 ± 0.6
LVIDs (mm)	3.9 ± 0.5	4.2 ± 0.4	3.9 ± 0.6	3.7 ± 0.3
FS (%)	15.3 ± 2.6	17.1 ± 3.8	14.7 ± 2.4	16.7 ± 2.7
LVAWd (mm)	1.8 ± 0.2	2.0 ± 0.2	1.9 ± 0.2	2.2 ± 0.3
LVAWs (mm)	3.3 ± 0.2	3.6 ± 0.4	3.5 ± 0.4	4.1 ± 0.3
LVPWd (mm)	2.1 ± 0.4	2.4 ± 0.6	2.3 ± 0.6	2.4 ± 0.4
LVPWs (mm)	3.6 ± 0.6	3.8 ± 0.6	3.9 ± 0.4	4.0 ± 0.6
E/A ratio	1.5 ± 0.1	1.8 ± 0.3	1.5 ± 0.3	2.0 ± 0.2
IVRT (ms)	26.1 ± 1.3	28.3 ± 3.9	24.5 ± 3.1	31.7 ± 3.5^*^

*Note*: Values are means ± SD. HFpEF‐CON, *n* = 10; HFpEF‐HEAT, *n* = 10. ^*^Significantly different from Pre within group. Abbreviations: CO, cardiac output; *E*/*A* ratio, ratio of peak early (*E*) to late (*A*) transmitral Doppler flow velocities; EF, ejection fraction; FS, fractional shortening; HFpEF, heart failure with preserved ejection fraction; IVRT, isovolumic relaxation time; LVAWd, left ventricular anterior wall thickness in diastole; LVAWs, left ventricular anterior wall thickness in systole; LVIDd, left ventricular internal diameter at end‐diastole; LVIDs, left ventricular internal diameter at end‐systole; LVPWd, left ventricular posterior wall thickness in diastole; LVPWs, left ventricular posterior wall thickness in systole; SV, stroke volume.

As detailed above and consistent with the echocardiography data presented in Table 5, the obese ZSF1 rat exhibits key features of HFpEF including preserved EF, diastolic dysfunction (e.g., elevated *E*/*A* ratio, prolonged IVRT) and cardiac remodelling (e.g., increased LVAWd and LVPWd) relative to their healthy counterparts (Belbis et al., 2025; Espino‐Gonzalez et al., 2021; Nguyen et al., 2020; Schauer et al., 2020; Stolina et al., 2020). As such, this model serves as a clinically relevant tool for translational research aimed at elucidating disease mechanisms and evaluating novel therapeutic strategies. While our previous studies demonstrated that 8 weeks of heat therapy exerted a protective effect on cardiac function in obese ZSF1 rats by preventing the progressive decline in left ventricular EF (Belbis et al., 2025), no differences in cardiac structure or function between HFpEF‐CON and HFpEF‐HEAT were observed in the present study (Table 5). The only exception was an elevation in IVRT post‐heat therapy, which warrants further investigation. A potential reason for this discrepancy may relate to differences in disease severity or progression between investigations, as the current animals appear to present with a more advanced HFpEF phenotype compared to those in our previous study, which showed higher EF (∼67–74%) and lower *E*/*A* ratio (∼1.3–1.6).

Similar to the findings for healthy rats, local noradrenaline had no effects on MAP or HR at rest or during contractions in HFpEF‐CON or HFpEF‐HEAT (Table 1). Moreover, the differences in MAP and HR between healthy and HFpEF groups can be ascribed to the anaesthetics used, as mentioned above. Isoflurane was utilized in HFpEF animals to minimize the potential confounding influence of dexmedetomidine on sympathetic regulation (Kenney et al., 2014). Notably, the presence of functional sympatholysis under dexmedetomidine in healthy rats indicates that the response is preserved, although its magnitude may have been underestimated due to the anaesthetic's sympatholytic properties.

In Part B, we hypothesized that functional sympatholysis would be impaired in HFpEF but ameliorated with heat therapy. As illustrated in Figures 4 and 5, the magnitude of sympatholysis was markedly reduced in HFpEF‐CON compared to healthy rats. This was the result of an exacerbated noradrenergic response at rest, which was unopposed during contractions. Although in general agreement with recent evidence, impaired functional sympatholysis in HFpEF patients appears to arise from diminished vascular responsiveness to sympathoexcitation at rest that remains unrestrained or excessive during exercise (Alpenglow et al., 2024, 2023). Of note, microneurography recordings in patients with HFpEF reveal elevated muscle sympathetic nerve activity (MSNA) at rest and during exercise (Badrov et al., 2025; Takeda et al., 2024; Washio et al., 2025). It has been postulated that reduced α‐adrenergic responsiveness may constitute a protective adaptation to chronically sympathetic overactivity in this disease (Alpenglow et al., 2024; Washio et al., 2025). Notwithstanding that the current preclinical model replicates the impaired functional sympatholysis reported in HFpEF patients, it is plausible that distinct clinical phenotypes, multiple comorbidities, medications and sex differences in vascular control may account, at least in part, for the contrasting responses observed in animal versus human data at rest.

As depicted in Figures 4 and 5, heat therapy attenuated the exaggerated noradrenergic vasoconstriction observed at rest in HFpEF rats, bringing their ${P_{O_{2}}}_{\mathrm{is}}$ response to noradrenaline in line with that of healthy controls. However, heat therapy had no effect on the ${P_{O_{2}}}_{\mathrm{is}}$ response to noradrenaline during muscle contractions, indicating that functional sympatholysis remained impaired in the HFpEF‐HEAT group compared to healthy controls and was not improved relative to HFpEF‐CON. These findings challenge our initial hypothesis but are nonetheless clinically relevant, as they demonstrate for the first time that heat therapy may blunt excessive sympathetic vasoconstriction at rest without affecting neurovascular control during contractions. In healthy older adults, 4 weeks of warm baths (40°C, 30 min/day, 5 days/week) significantly lowered resting MSNA burst frequency (Cui et al., 2022), while in obese women with polycystic ovary syndrome, 30 sessions of hot tub therapy over 8–10 weeks reduced resting MSNA and improved multiple cardiovascular risk markers (Ely et al., 2019). Importantly, the present study extends these findings by suggesting that heat therapy may also reduce noradrenergic responsiveness at the vascular level. Together, these observations raise the possibility of dual, complementary adaptations to heat therapy: a reduction in central sympathetic outflow and a decrease in peripheral vascular responsiveness to adrenergic stimulation.

### 4.3 Experimental considerations

Accumulating evidence supports that obese ZSF1 rats recapitulate key features of HFpEF, including preserved ejection fraction, impaired diastolic filling, skeletal muscle abnormalities, exercise intolerance, and comorbidities such as hypertension, obesity and diabetes (Horgan et al., 2014; Valero‐Muñoz et al., 2017). The obese ZSF1 rat has thus been employed widely in basic and applied studies targeting both cardiac and peripheral impairments in the setting of HFpEF. While no direct comparison of echocardiographic parameters between obese ZSF1 and healthy rats was performed in the current study, the data presented in Table 5 are consistent with those previously reported in this preclinical model of HFpEF (Espino‐Gonzalez et al., 2021; Leite et al., 2015; Nguyen et al., 2020; Schauer et al., 2020).

While lean ZSF1 rats share a genetic background with the obese model, their hypertensive and cardiometabolic abnormalities may limit their suitability as healthy controls (Büttner et al., 2025; Hamdani et al., 2013; Signore et al., 2021). Sprague–Dawley rats were thus chosen for Part A due to their normotensive, non‐diseased phenotype (cf. Signore et al., 2021) and consistency with our prior ${P_{O_{2}}}_{\mathrm{is}}$ studies.

The present study represents the first attempt to evaluate the effects of heat therapy on noradrenergic regulation of muscle ${P_{O_{2}}}_{\mathrm{is}}$ in a preclinical model of HFpEF. Only male ZSF1 rats were used herein to establish feasibility and lay the groundwork for subsequent investigations into sex‐specific responses. Future work should thus include female animals, particularly given the higher prevalence of HFpEF in women (Dunlay et al., 2017).

An important limitation of the study is that different anaesthetic protocols were used in healthy and HFpEF animals. As such, physiological inferences from comparisons between these groups should be made with caution. These comparisons are included to provide a frame of reference but do not represent the primary outcomes of the study. Moreover, given that healthy rats were ∼2–3 months old and the HFpEF rats were ∼6–7 months old at the time of ${P_{O_{2}}}_{\mathrm{is}}$ data collection, these groups fall within the young to middle‐aged adult range in rats, respectively. The age of the ZSF1 rats corresponds to the time point at which the HFpEF phenotype was established in this preclinical model and the intervention period has been completed.

Although repeated measures were not obtained herein, confidence intervals, individual response patterns, and responder analysis were used to evaluate the internal robustness of the main outcomes (i.e., Δ$P_{O_{2}}$/MAP and AUC/MAP). Blood pressure was only measured post‐treatment under anaesthesia; future studies should assess pre–post changes in conscious animals to further evaluate the cardiovascular effects of heat therapy.

### 4.4 Summary and conclusions

The present study is the first to examine the noradrenergic regulation of skeletal muscle ${P_{O_{2}}}_{\mathrm{is}}$ in healthy and HFpEF rats, as well as the impact of repeated heat therapy on those responses. Consistent with functional sympatholysis, muscle contractions blunted the reduction in spinotrapezius ${P_{O_{2}}}_{\mathrm{is}}$ with local noradrenaline compared to the resting condition in healthy rats. HFpEF rats exhibited exaggerated noradrenergic responses at rest, which remained unopposed during muscle contractions. Heat therapy failed to ameliorate functional sympatholysis in HFpEF rats but restored the noradrenergic response in resting skeletal muscle. These observations provide novel mechanistic insights into skeletal muscle dysfunction and plasticity in HFpEF and suggest that heat therapy might constitute a useful adjunct intervention for this patient population. Furthermore, the present results indicate that a novel combination of phosphorescence quenching (muscle ${P_{O_{2}}}_{\mathrm{is}}$) and superfusion techniques may be used to evaluate the impact of disease and treatment on functional sympatholysis in the anaesthetized rat.

## AUTHOR CONTRIBUTIONS

Conception and design: Edward T. N. Calvo, Bruno T. Roseguini, Igor A. Fernandes and Daniel M. Hirai. Data collection: Edward T. N. Calvo, Jacob M. Pontorno, Taciane M.M. Pejon, Michael D. Belbis and Daniel M. Hirai. Data analysis and interpretation: Edward T. N. Calvo, Jacob M. Pontorno, Benjamin Zeidler, Bruno T. Roseguini, Igor A. Fernandes and Daniel M. Hirai. Drafting manuscript: Edward T. N. Calvo and Daniel M. Hirai. Critical revision: all authors. All authors have read and approved the final version of this manuscript and agree to be accountable for all aspects of the work in ensuring that questions related to the accuracy or integrity of any part of the work are appropriately investigated and resolved. All persons designated as authors qualify for authorship, and all those who qualify for authorship are listed.

## CONFLICT OF INTEREST

None declared.

## Data Availability

The data that support the findings of this study are available from the corresponding author upon reasonable request.
